# Genome-wide identification and characterization of *Respiratory Burst Oxidase Homolog* genes in six Rosaceae species and an analysis of their effects on adventitious rooting in apple

**DOI:** 10.1371/journal.pone.0239705

**Published:** 2020-09-25

**Authors:** Chenxia Cheng, Qinqin Che, Shenghui Su, Yuan Liu, Yongzhang Wang, Xiaozhao Xu

**Affiliations:** 1 College of Horticulture, Qingdao Agricultural University, Qingdao, China; 2 Qingdao Key Laboratory of Genetic Development and Breeding in Horticultural Plants, Qingdao Agricultural University, Qingdao, China; 3 Qingdao Key Lab of Modern Agriculture Quality and Safety Engineering, Qingdao Agricultural University, Qingdao, China; 4 Laixi Elite Cultivars Propagation Farm, Laixi, Qingdao, China; University of Helsinki, FINLAND

## Abstract

Adventitious root formation is essential for plant propagation, development, and response to various stresses. Reactive oxygen species (ROS) are essential for adventitious root formation. However, information on Respiratory Burst Oxidase Homolog (RBOH), a key enzyme that catalyzes the production ROS, remains limited in woody plants. Here, a total of 44 *RBOH* genes were identified from six Rosaceae species (*Malus domestica*, *Prunus avium*, *Prunus dulcis '*Texas’, *Rubus occidentalis*, *Fragaria vesca* and *Rosa chinensis*), including ten from *M*. *domestica*. Their phylogenetic relationships, conserved motifs and gene structures were analyzed. Exogenous treatment with the RBOH protein inhibitor diphenyleneiodonium (DPI) completely inhibited adventitious root formation, whereas exogenous H_2_O_2_ treatment enhanced adventitious root formation. In addition, we found that ROS accumulated during adventitious root primordium inducing process. The expression levels of *MdRBOH-H*, *MdRBOH-J*, *MdRBOH-A*, *MdRBOH-E1* and *MdRBOH-K* increased more than two-fold at days 3 or 9 after auxin treatment. In addition, *cis*-acting element analysis revealed that the *MdRBOH-E1* promoter contained an auxin-responsive element and the *MdRBOH-K* promoter contained a meristem expression element. Based on the combined results from exogenous DPI and H_2_O_2_ treatment, spatiotemporal expression profiling, and *cis*-element analysis, *MdRBOH-E1* and *MdRBOH-K* appear to be candidates for the control of adventitious rooting in apple.

## 1. Introduction

Adventitious rooting is essential for both woody perennial plants and herbaceous plants. Indeed, adventitious root formation is the cornerstone of woody perennial plant propagation, in which new plants are propagated vegetatively from elite genotypes [1]. Monocot plants such as cereals generate numerous crown roots, a type of adventitious root that dominates their root system [2]. Adventitious roots are also induced to permit survival under oxygen-deficient conditions in plants that are adapted to waterlogging and submersion [3, 4]. In recent years, our understanding of the mechanisms of adventitious root formation has improved greatly, especially in herbaceous plants for which adventitious rooting can be induced easily [5]. However, information on adventitious rooting is still lacking for many recalcitrant plants such as apple rootstocks [6–13]. More research on adventitious root formation will enrich our basic knowledge and aid in efforts to optimize propagation conditions for better rooting of recalcitrant species.

An increasing number of studies indicate that homeostasis and signaling of endogenous hormones are important for adventitious root formation [14, 15]. It has become clear that auxin plays a central role in adventitious root formation [16]. The other phytohormones, including cytokinin (CK), ethylene (ETH), gibberellic acid (GA), strigolactones (SLs), abscisic acid (ABA), salicylic acid (SA), brassinosteroids (BRs), and jasmonate (JA), have been shown to influence adventitious root development directly, by interacting with one another, or by interacting with auxin [5].

In addition, various small-molecule compounds such as nitric oxide (NO), hydrogen gas (H_2_), hydrogen sulfide (H_2_S), methane (CH_4_) and hydrogen peroxide (H_2_O_2_), are also involved in adventitious root formation and development [17]. According to the present evidence, H_2_O_2_ mainly acts to stimulate adventitious root formation. Treatment with 20–40 μM exogenous H_2_O_2_ significantly enhanced the adventitious rooting ability of cucumber [18], and 200 μM H_2_O_2_ increased adventitious root length and number in marigold and chrysanthemum [19, 20]. Moreover, it has been demonstrated that both calcium and calmodulin are two downstream signaling molecules in adventitious rooting induced by H_2_O_2_ in marigold [21]. In addition, H_2_O_2_ accumulated in cucumber plants during adventitious root formation [3]. Scavenging endogenous H_2_O_2_ through the application of diphenyleneiodonium (DPI), which inhibits the generation of reactive oxygen species (ROS) by RBOH decreased the number of adventitious root in cucumber and chrysanthemum [3, 20]. RBOH is a key enzyme that catalyzes the production of O_2_^−^, which is rapidly transformed in the plant into H_2_O_2,_ a more stable form of ROS [22]. In general, information on the function of ROS produced by RBOHsin adventitious root formation is not as comprehensive as our understanding of their roles in plant stress response [23–26], root hair development [27, 28], main root growth [29–31] and lateral root development [32–34]. The expression levels of *RBOH1* and *RBOH3* increased more than 2.5-fold during adventitious root formation triggered by waterlogging in wheat [4]. Similarly, the expression levels of *CsRBOHB* and *CsRBOHF3* were enhanced by ethylene and auxin, ultimately leading to adventitious root formation in cucumber [3]. These results indicate that RBOH, also known as NADPH oxidase, may play an important role in adventitious root formation. However, a role for *RBOH* family genes in adventitious rooting has not been reported in apple.

Here, we used bioinformatics methods to identify *RBOH* genes in the genomes of six Rosaceae species: *Malus domestica*, *Prunus avium*, *Prunus dulcis ‘*Texas’, *Rubus occidentalis*, *Fragaria vesca*, and *Rosa chinensis*. We analyzed their phylogenetic relationships, gene structures, conserved motifs and domains, and chromosome locations. We also analyzed the expression of apple *MdRBOHs* during adventitious rooting and used exogenous H_2_O_2_ and DPI treatments to assess the potential effects of RBOH on adventitious rooting. This study presents the molecular characteristics of *RBOH* genes from six Rosaceae species. It adds to our understanding of RBOH function in adventitious root formation and lays a foundation for optimizing parameters for better rooting of apple rootstocks.

## 2. Materials and methods

### 2.1. Identification and classification of RBOH genes

Sequences and genome-related information for *M*. *domestica* (apple), *P*. *avium* (sweet cherry), *P*. *dulcis ‘*Texas’ (almond), *R*. *occidentalis* (black raspberry), *F*. *vesca* (strawberry) and *R*. *chinensis* (rose) were downloaded from the Genome Database for Rosaceae (GDR, https://www.rosaceae.org/). Arabidopsis RBOH protein sequences were obtained from The Arabidopsis Information Resource (TAIR, https://www.arabidopsis.org/).

Using the Arabidopsis RBOH amino acid sequences as queries, candidate RBOHs were identified from the genome databases using two basic local alignment search tool (BLAST) methods implemented in TBtools (https://github.com/CJ-Chen/TBtools). After removing repetitive and redundant sequences, primary RBOH proteins were identified using BLAST methods in the UniProKB/Swiss-Prot database. Their conserved domains were analyzed using CD-search at the NCBI website (https://www.ncbi.nlm.nih.gov/Structure/cdd/wrpsb.cgi) to determine whether each candidate sequence contained an NADPH_Ox (PF08414) domain.

The properties of each RBOH protein sequence, including its length (aa), molecular weight (MW), isoelectric point (pI) and predicted subcellular localization, were calculated using the ExPASy-Compute pI/MW tool (http://web.expasy.org/compute_pi/) and Plant-mPLoc (http://www.csbio.sjtu.edu.cn/bioinf/plant-multi/#).

### 2.2. Phylogenetic analysis

A species phylogenetic tree was obtained from the Common Taxonomy Tree (https://www.ncbi.nlm.nih.gov/Taxonomy/CommonTree/wwwcmt.cgi) and visualized using EvolView (http://www.evolgenius.info/evolview/). Phylogenetic trees were constructed from RBOH protein sequences using MEGA7.0 software (Arizona State University, Tempe, AZ, USA) with the Maximum Likelihood method and 1000 bootstrap replicates [35].

### 2.3. Chromosomal distribution and synteny analysis

All *RBOH* genes were mapped to their respective chromosomes using Amazing Gene Location from GTF/GFF in TBtools. Synteny blocks within the apple genome and between the apple and Arabidopsis genomes were constructed using Quick MCScanX Wrapper and were visualized using the Dual Synteny Plotter in TBtools.

### 2.4. Sequence analysis

Motifs in RBOH proteins were identified using the MEME suite (http://meme-suite.org/). Conserved domains within the RBOH proteins were identified using Batch CD-Search (https://www.ncbi.nlm.nih.gov/cdd). The structures of the *RBOH* genes were constructed using the Amazing Optional Gene Viewer in TBtools. The *cis*-elements present in all promoter sequences (−2000 bp upstream) were identified using PlantCARE [36].

### 2.5. Plant materials and treatments

Stem cuttings of ‘Gala’ apples were sub-cultured in a normal medium of Murashige and Skoog (MS), 7.5 g L^−1^ agar and 30 g L^−1^ sugar (pH 5.8) with 0.5 mg L^−1^ IBA and 0.2 mg L^−1^ 6-BA under a 16 h light/8 h dark photoperiod with day/night temperatures of 25±1°C and 20±1°C. When stem cuttings had grown to 2–3 cm in length, they were transferred into rooting medium containing 1/2 MS, 30 g L^−1^ sugar, 7.5 g L^−1^ agar, 0.5 mg L^−1^ IBA and 0.1 mg L^−1^ NAA (pH 5.8) [37]. For adventitious rooting assays, stem cuttings were transferred into rooting medium supplemented with various concentrations of H_2_O_2_ (1 mM, 5 mM and 25 mM) (Sigma–Aldrich, St. Louis, MO, USA) and DPI (5 μM, 10 μM and 20 μM) (Sigma–Aldrich, St. Louis, MO, USA). There were three biological replicates for each treatment, and each biological replicate consisted of six tissue culture cuttings.

Apple tissues and organs, including young roots, stems, leaves, and shoot apices, were harvested from two-year-old own-rooted ‘Gala’ seedlings grown in the field.

### 2.6. Histological analysis

We collected 0.5 cm sections of ‘Gala’ stem cuttings at 0, 6, 9, and 12 d after subculture on rooting medium. The samples were fixed in a 50:5:5 (v/v/v) ethanol/formaldehyde/acetic acid (FAA) solution for 2 days at room temperature. Then the sections were dehydrated in an ethanol series (50%, 70%, 85%, 95%, and 100%), infiltrated with xylene, and embedded in paraffin. Longitudinal sections with a thickness of 10 μm were cut with a rotary microtome (KD-2258, KEDEE, China) and stained with toluidine blue [1]. For nitro-blue-tetrazolium (NBT) staining of apple stem cuttings, the cross sections were cut after the staining according to the method described previously [3].

### 2.7. Gene expression analysis

For relative expression analysis of *RBOH* genes during adventitious rooting, 0.5 cm sections of ‘Gala’ stem cuttings were frozen in liquid nitrogen at 0, 3, 6, and 9 d after subculture on rooting medium. Total RNA was extracted from approximately 0.5 g of frozen sample using the TIANGEN Plant RNA Kit (TIANGEN biotech CO., LTD, Beijing, China, DP305). Then 1 μg of total RNA was used for first-strand cDNA synthesis using the PrimeScript™ RTase (TaKaRa Biotechnology, Dalian, China). Quantitative RT-PCR was performed using SYBR green reagents (RR820A, Takara, Dalian, China) on an Applied Biosystems 7500 real-time PCR system. The apple *EF1α* gene was used as the control. Relative expression levels were calculated according to the 2^−ΔΔCT^ method [38]. Three independent biological replicates and technical replicates were conducted. Primer sequences used in this study are listed in S1 Table.

## 3. Results

### 3.1. Identification and characterizaition of RBOH family genes

BLASTp were conducted in the genome databases of six Rosaceae species using the protein sequence of AtRBOHs as the query sequences. After removing repetitive and redundant sequences, a total of 52 *RBOH* genes were originally identified using BLAST methods in the UniProKB/Swiss-Prot database (S1 Table). Based on the presence of apparently complete NADPH_Ox (PF08414) domain and EF-hand (CD00051), 44 genes were subsequently selected and annotated as being *RBOH* genes. Genes without complete predicted NADPH_Ox and EF-hand domains were removed (MD12G1213800, Pav_co4068891.1, Pav_sc0000671.1, Pav_sc0001450.1, Prudul26A013915P2, Prudul26A019458P1, RcHm_v2.0_Chr2g0103591, RcHm_v2.0_Chr2g0103601) (S2 Table). Finally, a total of 44 candidate *RBOHs* were identified from six Rosaceae genomes: ten from *M*. *domestica* (*MdRBOH*), four from *P*. *avium* (*PavRBOH*), seven from *P*. *dulcis ‘*Texas’ (*PdRBOH*), seven from *R*. *occidentalis* (*RoRBOH*), seven from *F*. *vesca* (*FvRBOH*) and nine from *R*. *chinensis* (*RcRBOH*). The *RBOHs* were named based on their phylogenetic relationships with their Arabidopsis counterparts (Table 1). The deduced RBOH protein sequences ranged from 758 (RoRBOH-K) to 972 (PdRBOH-D) amino acids in length. Their molecular weights and pIs ranged from 86.88 (RcRBOH-A2) to 109.59 (PavRBOH-F) kDa and from 7.59 (MdRBOH-H) to 9.35 (PdRBOH-F), respectively (Table 1). Subcellular localization predications indicated that the ROBHs were all located in the cell membrane (Table 1). In addition, transiently expressed MdRBOH-E1_GFP or MdRBOH-F1/F2_GFP, or MdRBOH-J_GFP fusion protein in tobacco leaves supporting the notion that RBOHs are localized to the cell membrane (S1 Fig).

**Table 1:** RBOH genes identified in this study. Subcellular localization predications indicated that the ROBHs were all located in the cell membrane.

Species	Gene name	Locus	chromosome	start position	end position	Length (aa)	MW (kD)
*Malus domestica*	*MdRBOH-D2*	MD16G1145100	Chr16	11156971	11161436	965	108.64
	*MdRBOH-D1*	MD13G1134500	Chr13	10304950	10310061	962	108.44
	*MdRBOH-A*	MD06G1128500	Chr06	27087364	27094696	941	105.38
	*MdRBOH-F1*	MD06G1093000	Chr06	22375873	22385466	961	109.27
	*MdRBOH-F2*	MD14G1113700	Chr14	18347131	18359289	959	109.37
	*MdRBOH-E1*	MD02G1099900	Chr02	7918841	7925357	912	103.16
	*MdRBOH-E2*	MD15G1222800	Chr15	18082033	18086949	946	106.97
	*MdRBOH-H*	MD11G1118400	Chr11	10784434	10788882	859	98.11
	*MdRBOH-J*	MD03G1106300	Chr03	9106580	9110548	823	93.42
	*MdRBOH-K*	MD14G1211700	Chr14	29690183	29695068	785	89.50
*Prunus avium*	*PavRBOH-A*	Pav_sc0000589	Chr05	10841240	10843909	943	105.4
	*PavRBOH-F*	Pav_sc0000886	Chr05	8458927	8463180	964	109.59
	*PavRBOH-E2*	Pav_sc0000129	Chr07	15367462	15367766	799	90.27
	*PavRBOH-H*	Pav_sc0000323	Chr06	5597348	5598268	860	98.43
*Prunus dulcis Texas*	*PdRBOH-A*	Prudul26A009078P1	Chr05	12324110	12329280	942	105.27
	*PdRBOH-D*	Prudul26A009232P1	Chr01	19719419	19724815	972	108.95
	*PdRBOH-B*	Prudul26A005532P1	Chr06	27292497	27296750	894	101.9
	*PdRBOH-E*	Prudul26A013915P1	Chr07	17253193	17258382	965	103.46
	*PdRBOH-F*	Prudul26A017061P1	Chr05	10368633	10377258	913	109.55
	*PdRBOH-H*	Prudul26A019458P2	Chr06	6163889	6168519	857	98.23
	*PdRBOH-K*	Prudul26A030363P1	Chr05	16131323	16146293	811	92.5
*Rubus occidentalis*	*RoRBOH-J*	Ro03_G02103	Chr03	7378990	7384860	841	95.98
	*RoRBOH-E*	Ro01_G03041	Chr01	4216430	4224173	925	105.10
	*RoRBOH-F*	Ro05_G16791	Chr05	7260878	7272779	957	109.06
	*RoRBOH-K*	Ro05_G03331	Chr05	2246266	2252031	758	86.94
	*RoRBOH-A*	Ro05_G01806	Chr05	4074465	4082558	919	103.69
	*RoRBOH-D*	Ro04_G08892	Chr04	7473145	7479394	933	105.15
	*RoRBOH-B*	Ro06_G18529	Chr06	27530616	27538049	862	98.80
*Fragaria vesca*	*FvRBOH-A*	FvH4_5g10010.1	Chr05	5700687	5708036	917	103.18
	*FvRBOH-D*	FvH4_4g22100.1	Chr04	25038971	25043196	935	105.46
	*FvRBOH-B*	FvH4_6g06490.1	Chr06	3796200	3801416	879	100.64
	*FvRBOH-E*	FvH4_1g09270.1	Chr01	4945413	4949975	867	97.94
	*FvRBOH-F*	FvH4_5g03310.1	Chr05	1974927	1983225	945	107.43
	*FvRBOH-H*	FvH4_3g34420.1	Chr03	29860660	29865289	865	98.37
	*FvRBOH-K*	FvH4_5g13850.1	Chr05	7816138	7820214	818	93.12
*Rosa chinensis*	*RcRBOH-A1*	RcHm_v2.0_Chr7g0188251	Chr07	7870102	7877173	925	103.87
	*RcRBOH-A2*	RcHm_v2.0_Chr7g0202531	Chr07	20116816	20123208	766	86.88
	*RcRBOH-D*	RcHm_v2.0_Chr4g0428361	Chr04	53408287	53412982	936	105.85
	*RcRBOH-B1*	RcHm_v2.0_Chr3g0455621	Chr03	5279721	5284973	878	101.05
	*RcRBOH-B2*	RcHm_v2.0_Chr3g0455641	Chr03	5311755	5316513	881	101.46
	*RcRBOH-E*	RcHm_v2.0_Chr2g0095831	Chr02	8765834	8770818	923	104.91
	*RcRBOH-F*	RcHm_v2.0_Chr7g0196471	Chr07	14440777	14448994	953	108.37
	*RcRBOH-H*	RcHm_v2.0_Chr5g0062021	Chr05	67592381	67596621	847	97.08
	*RcRBOH-K*	RcHm_v2.0_Chr7g0183081	Chr07	4122936	4127024	790	90.48

### 3.2. Phylogenetic and comparative analysis of RBOH genes in various plant species

**Fig 1:**
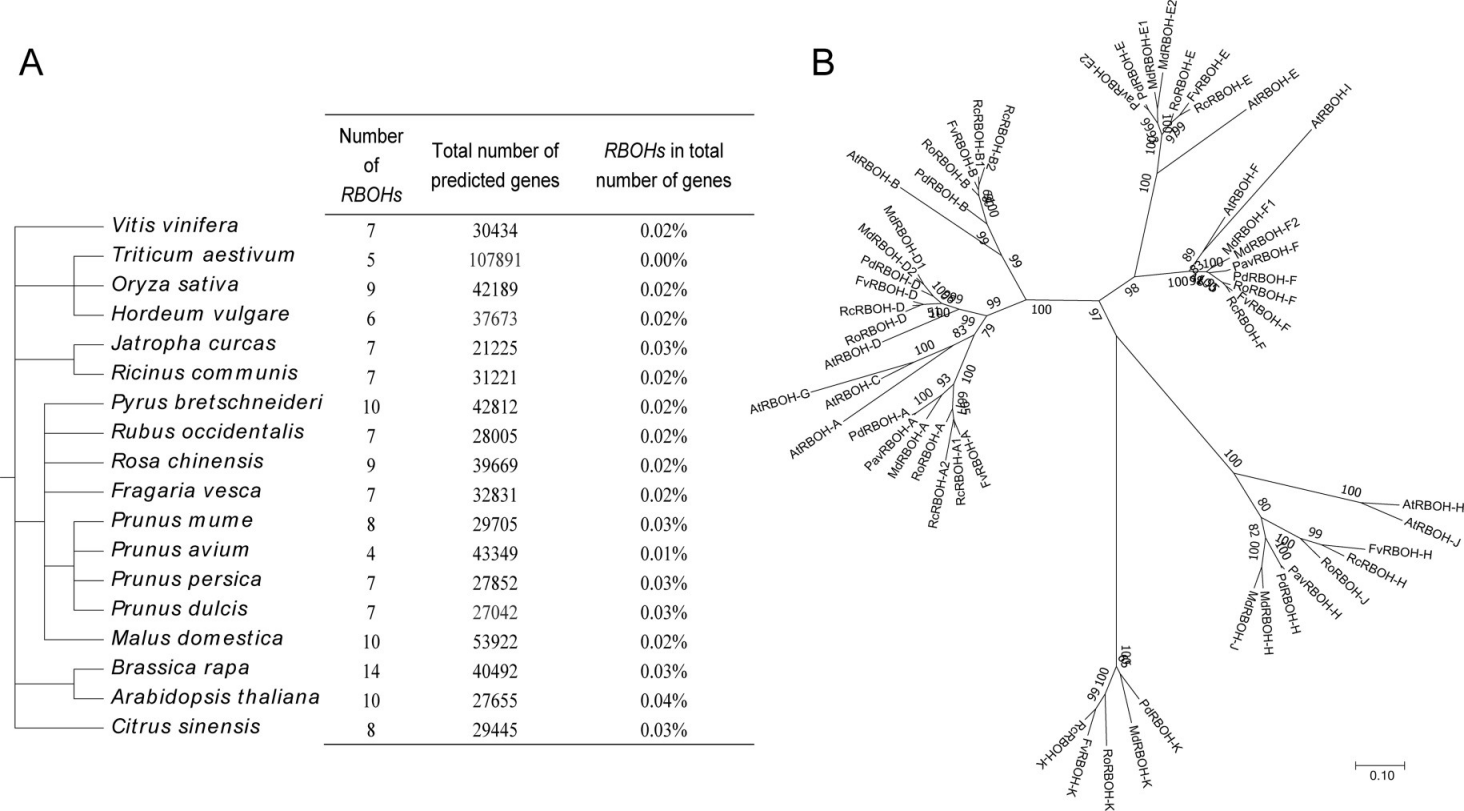
Phylogenetic and comparative analysis of *RBOH* genes from various plants. (A) A summary of the species phylogeny and the number of *RBOH* genes in each species. (B) Phylogenetic analysis of RBOH proteins from six Rosaceae species and Arabidopsis.

To investigate the evolutionary history of the *RBOH* family, we compared the number of *RBOHs* among 18 plant species [39–44]. As shown in Fig 1A, no individual species had more than 14 *RBOH* members. The percentage of *RBOHs* with respect to the total number of genes in each genome was low and differed little among species. This indicates that the number of the *RBOH* gene family in these 18 species was not associated with an increase in the total number of predicted genes.

To further evaluate the evolutionary relationships among RBOH family members from the six Rosaceae species and Arabidopsis, 54 RBOH predicted protein sequences were used to construct a phylogenetic tree (Fig 1B). The RBOHs clustered into seven subfamilies, which are indicated by different colors in Fig 1B. With the exception of the RBOH-K subfamily, the other six subfamilies contained at least one AtRBOH member. In addition, multiple sequence alignments of the 54 RBOH proteins were performed (S2 Fig). It can be found that the amino acid sequences of *RBOH* genes identified in this study and *AtRBOH* family members are highly conserved.

### 3.3. Chromosomal distribution and duplication of RBOH genes

**Fig 2:**
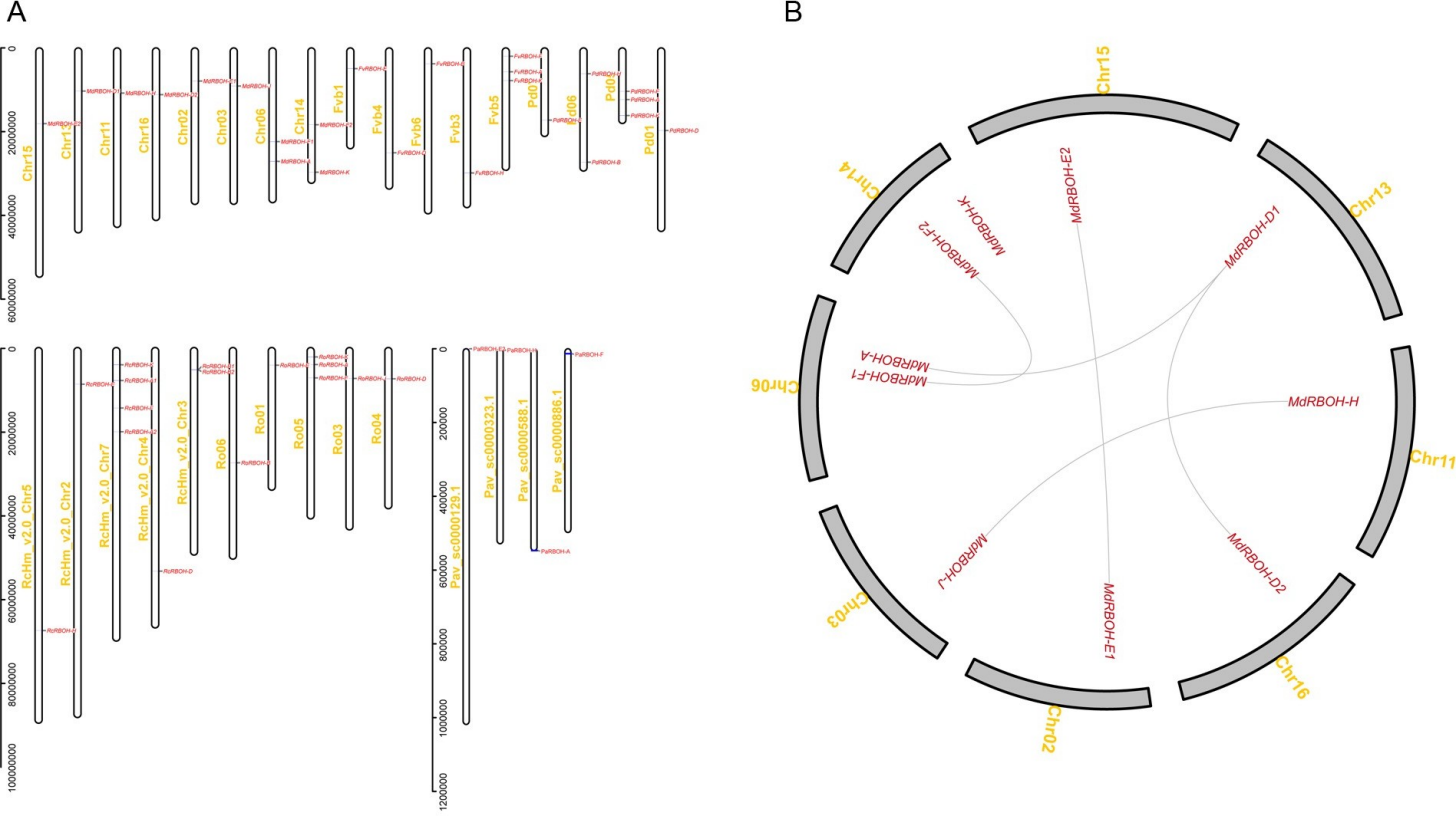
Chromosomal distribution and duplication of *RBOH* genes. (A) Chromosomal distribution of the *RBOH* genes among six Rosaceae species. (B) Synteny analysis of the apple *RBOH* genes. Syntenic apple *RBOH* genes are linked by lines.

The genomic distribution of the 44 *RBOH* genes is shown in Fig 2A. Only one tandem duplication event was identified according to the methods of Holub [45]. It occurred in the *Rosa chinensis RBOH* family, and the tandem duplicated gene pair was *RcRBOH-B1* and *RcRBOH-B2*. The other 42 *RBOH* genes were irregularly distributed on their species’ chromosomes, and no gene clusters were found. In apple, the 10 *MdRBOHs* were mapped to 8 of the 17 chromosomes. Seven *FvRBOHs*, seven *PdRBOHs*, nine *RcRBOHs*, seven *RoRBOHs* and four *PaRBOHs* were located on five strawberry chromosomes, four almond chromosomes, five rose chromosomes, five black raspberry chromosomes, and four sweet cherry chromosomes, respectively.

The synteny analysis (Fig 2B) and phylogenetic tree (Fig 1B) indicated that five gene duplication events had occurred in the apple *RBOH* family. The duplicated gene pairs were *MdRBOH-D1* and *MdRBOH-D2*, *MdRBOH-D1* and *MdRBOH-A*, *MdRBOH-E1* and *MdRBOH-E2*, *MdRBOH-F1* and *MdRBOH-F2*, and *MdRBOH-H* and *MdRBOH-J*.

### 3.4. Motif composition, conserved domains and gene structures of RBOH genes

**Fig 3:**
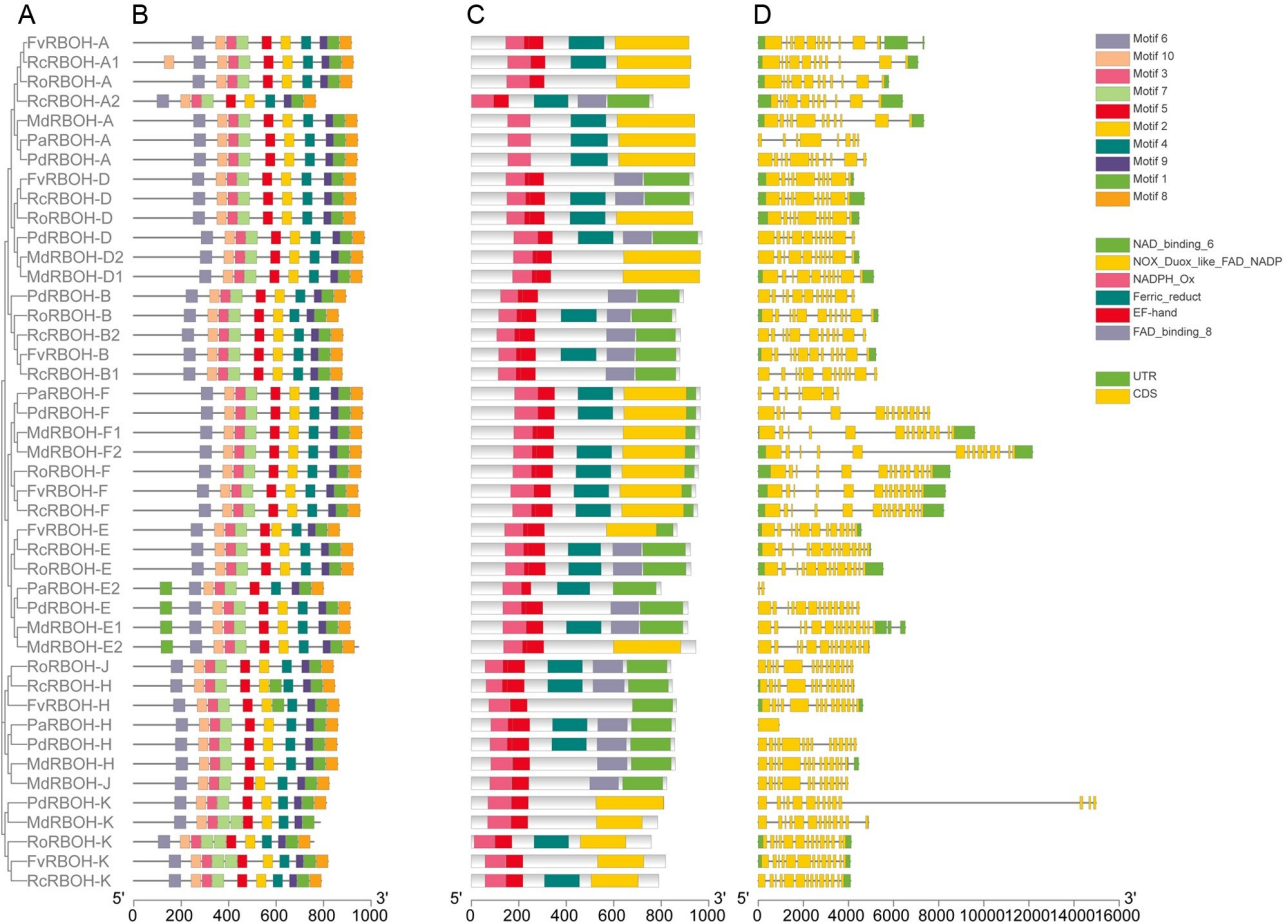
Phylogenetic relationships, motif composition, conserved domains, and gene structures of RBOH proteins and their corresponding genes from six Rosaceae species. (A) The phylogenetic tree was drawn based on the full-length protein sequences of RBOHs using MEGA 7.0. (B) Motifs in RBOH proteins. Ten motifs are indicated by different colored boxes. (C) Conserved domains of the RBOH proteins. Six conserved domains are indicated with different colored boxes. (D) Gene structures of the *RBOHs*. Introns, exons and untranslated regions (UTRs) are indicated by gray lines, yellow rectangles and green rectangles, respectively.

MEME analysis revealed ten conserved motifs within the 44 RBOH proteins (Fig 3B). In general, these ten motifs were present in all 44 RBOH proteins with a similar order and distribution. However, the N-terminal domain of the PaRBOH-E2, PdRBOH-E, MdRBOH-E1, and MdRBOH-E2, which belong to the same subfamily, all contains an additional motif 1. These results indicated that differences in the composition of the N-terminal domain may confer different biological functions to this subfamily.

We further analyzed the conserved domains of the 44 RBOHs (Fig 3C). They included NAD_binding_6 (pfam08030), NOX_Duox_like_FAD_NADP (CD06186), NADPH_Ox (pfam08414), Ferric_reduct (pfam01794), EF-hand (CD00051) and FAD_binding_8 (pfam08022) domains. Every RBOH contained the NADPH_Ox and EF-hand domains, which are characteristic of plant RBOH proteins (Fig 3C).

Gene structure analysis showed that the *RBOHs* had 1 to 14 exons and that members of the same subfamily shared the same or similar intron–exon patterns (Fig 3D). *RBOH* exons also tended to be quite short. In general, *RBOHs* in the same subfamily exhibited similar conserved domains, intron–exon organizations, and motif characteristics, which supports their classification in the same subfamily and their close evolutionary relationship.

### 3.5. Effects of DPI on auxin-induced adventitious root formation

**Fig 4:**
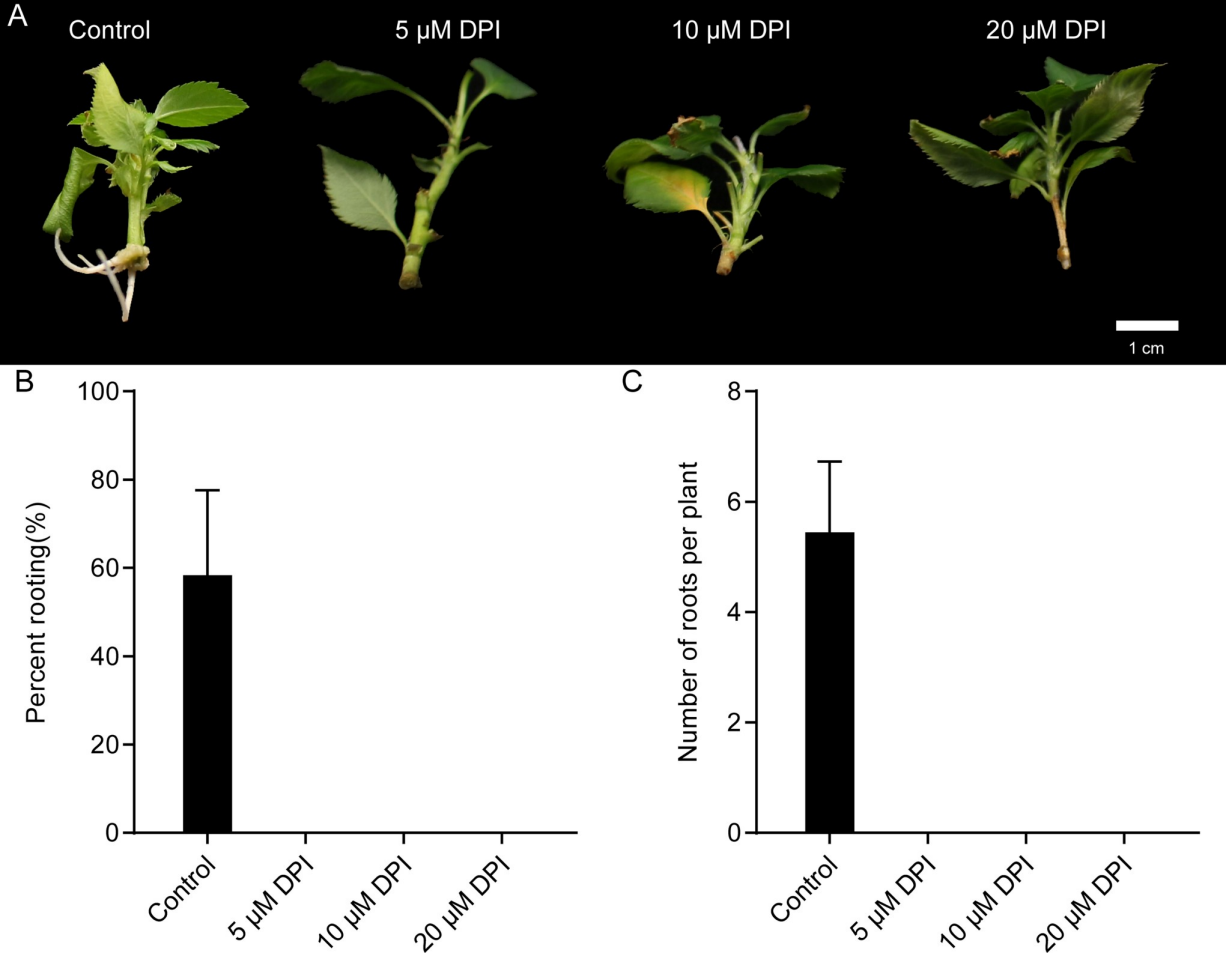
Effects of DPI on adventitious root formation of apple stem cuttings. (A) Adventitious root formation in apple stem cuttings after 18 days in rooting medium (control) or rooting medium supplemented with DPI (5 μM, 10 μM, and 20 μM). Percent rooting (B) and adventitious root number per cutting (C) were counted at 18 days. Bars indicate mean and Standard Deviation (SD) of three biological replicates; *n* = 6 individuals per replicate.

To investigate the potential role of RBOHs in apple adventitious root formation, DPI, which inhibits the generation of reactive oxygen species (ROS) by RBOH, was added to the rooting medium. After 18 days of treatment, adventitious roots were counted. Treatment of apple with DPI suppressed adventitious root formation (Fig 4). Treatment with 5 μM DPI completely suppressed adventitious root formation but did not damage the stem cuttings. On the other hand, treatment with 10 or 20 μM DPI completely suppressed adventitious root formation and also damaged the stem cuttings. These results indicate that the production of ROS by RBOH plays an important role in auxin-induced adventitious root formation in apple.

### 3.6. Effects of exogenous H2O2 on apple adventitious root formation

**Fig 5:**
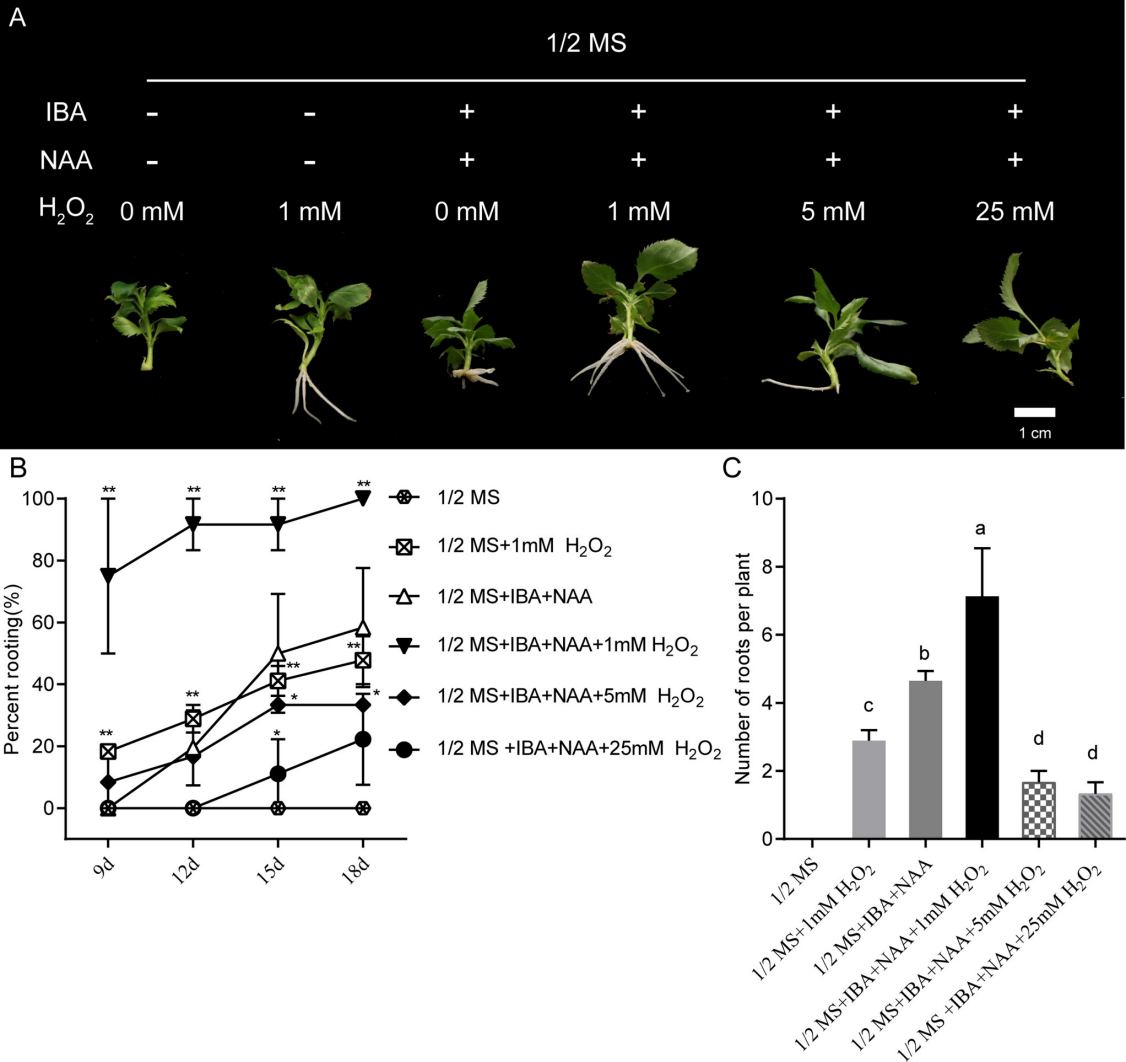
Effects of exogenous H_2_O_2_ on adventitious root formation of apple stem cuttings. (A) Adventitious root formation in apple stem cuttings after 18 days in rooting medium or rooting medium supplemented with H_2_O_2_ (1 mM, 5 mM, and 25 mM). “+” represents presence, “−” represents absence. The concentrations of IBA and NAA were 0.5 mg L^−1^ and 0.1 mg L^−1^, respectively. (B) Percent rooting. Bars indicate mean and SE from three biological replicates; *n* = 6 individuals per replicate. Asterisks indicate significant differences between stem cuttings subcultured in 1/2 MS and stem cuttings subcultured in 1/2 MS + IBA +NAA (Student’s *t*-test, **P < 0.01, *P < 0.05). (C) Adventitious root number per cutting. Adventitious root number per cutting was counted at 18 days. Bars indicate mean and SE from three biological replicates; *n* = 6 individuals per replicate. The statistical analysis was conducted using Duncan’s multiple range test (*P* < 0.05). The different lowercase letters indicate significant differences.

As shown in Fig 4, inhibiting the generation of ROS by the application of DPI completely suppressed adventitious root formation. Therefore, we next applied exogenous H_2_O_2_ to assess its effects on adventitious root formation. Various concentrations of H_2_O_2_ were applied to apple stem cuttings grown on auxin-free 1/2 MS medium or on 1/2 MS medium supplemented IBA and NAA. Exogenous H_2_O_2_ affected apple adventitious root formation in a dose-dependent manner (Fig 5). Treatment of apple stem cuttings with 1 mM H_2_O_2_ significantly enhanced adventitious rooting, but treatment with 5 mM or 25 mM H_2_O_2_ significantly inhibited adventitious root formation (Fig 5A). Treatment with 1 mM H_2_O_2_ not only caused significantly faster rooting, but also significantly increased adventitious root number compared with the other treatments (Fig 5B and 5C). Interestingly, 1 mM H_2_O_2_ treatment rescued the adventitious rooting defect of stem cuttings that were grown on auxin-free 1/2 MS medium (Fig 5).

### 3.7. Superoxide accumulation during adventitous root formation

**Fig 6:**
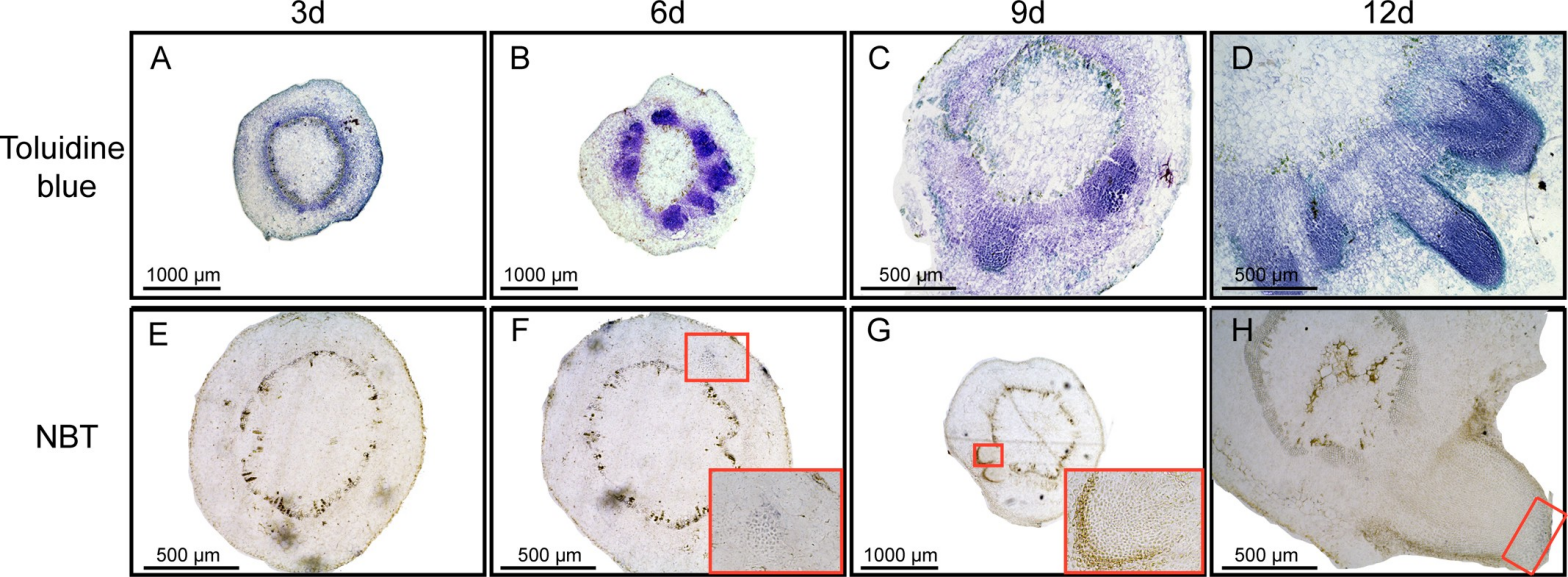
Reactive oxygen species are participated in adventitious root formation in apple stem cuttings. Stem cross-sections of ‘Gala’ apples taken at 3, 6, 9, and 12 days after subculture on rooting medium and stained with toluidine blue (A-D) and Nitro blue tetrazolium (NBT; O_2_^-^) (E-H), respectively. Scale bars (1000 μm, 500 μm, and 200 μm) are indicated in the figures. Pictures in red boxes show a 20⊆ view of the stem cuttings in (F, G). The rectangle indicates NBT-stained root tip in (H).

Cross sections of toluidine blue-stained stem cuttings showed that dome-shaped adventitious primordia were clearly visible in stem cross-sections at 6–9 days (Fig 6B and 6C). After 12 days, adventitious roots began to appear (Fig 6D). After 6 days, the amounts of O_2_^-^ (as shown by NBT) accumulated mainly in the differentiating adventitious root primordia (Fig 6F). After 9 days, the O_2_^-^ production was almost undetected when adventitious root primordia were completely differentiated (Fig 6G). Interestingly, the amounts of O_2_^-^ accumulated in the root tip during the adventitious root growth after 12 days (Fig 6H).

### 3.8. Expression patterns of MdRBOH genes during adventitous root formation

**Fig 7:**
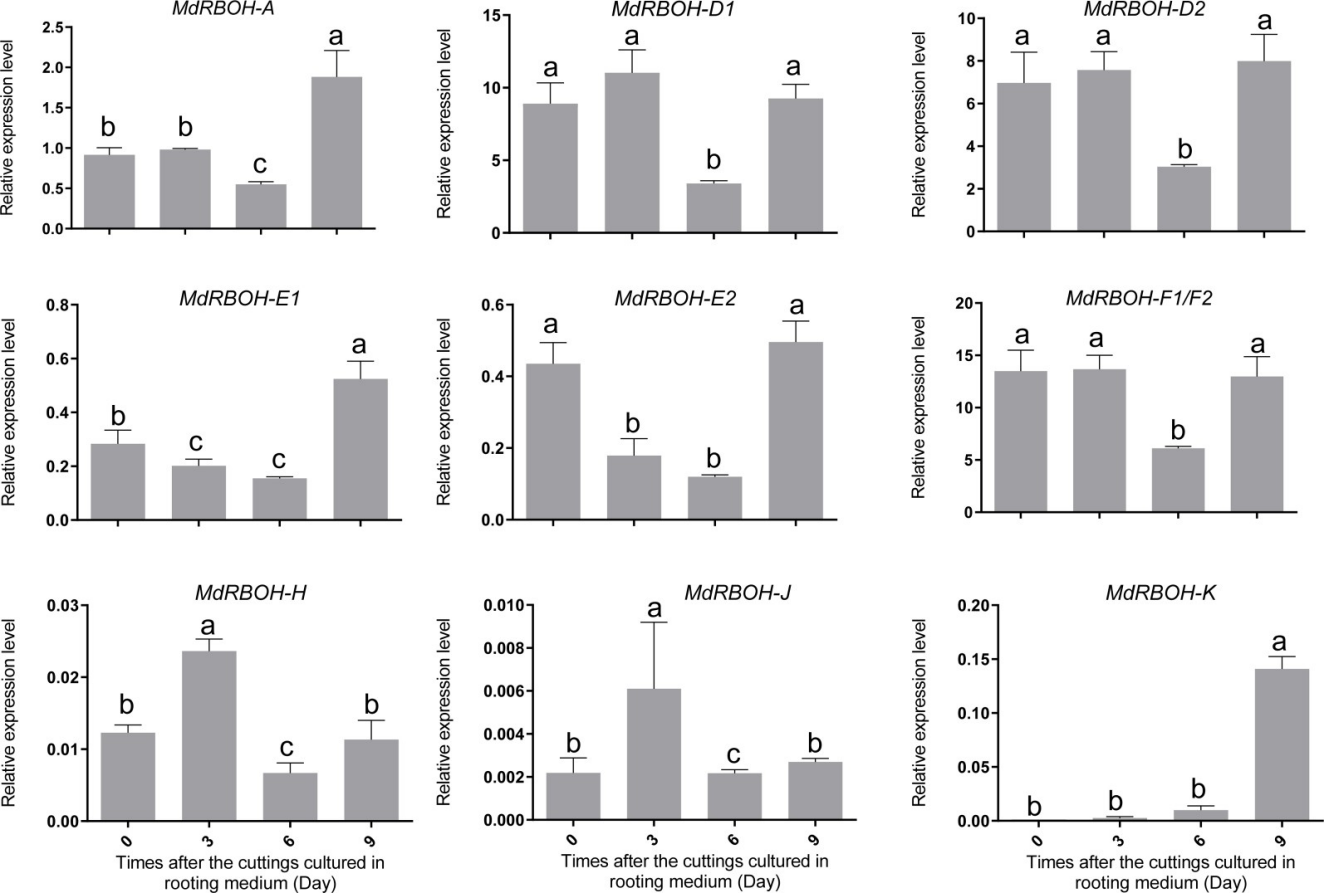
Relative expression levels of *MdRBOH* genes during the adventitious rooting process. Relative transcript levels of 10 *MdRBOH* genes were measured in stem tissue after the cuttings had been transferred to rooting medium for 0, 3, 6, or 9 days. Transcript levels were measured by qRT-PCR using apple *EF1α* as the reference gene. Data are means ± SD of three biological replicates. Refer to S1 Table for primers. The statistical analysis was conducted using Duncan’s multiple range test (*P* < 0.05). The different lowercase letters indicate significant differences.

To investigate whether *MdRBOH* genes are involved in apple adventitious root formation, we examined the expression profiles of ten *MdRBOH* genes during the induction of adventitious rooting. According to the histological features in Fig 6A–6D, we used stem cuttings collected at 0, 3, 6, and 9 days after sub-culturing in rooting medium for gene expression analysis. As shown in Fig 7, the expression of *MdRBOH-H* and *MdRBOH-J* increased more than two-fold on day 3 relative to day 0. In addition, the expression of *MdRBOH-A*, *MdRBOH-E1* and *MdRBOH-K* increased more than two-fold on day 9 relative to day 0. Of these five *RBOH* genes, *MdRBOH-A*, *MdRBOH-E1*, and *MdRBOH-K* showed an expression pattern that most closely tracked the appearance of adventitious root primordia. These results suggest that *MdRBOH-A*, *MdRBOH-E1*, and *MdRBOH-K* may be involved in the requirement for ROS generation during adventitious root formation in apple.

## 4. Discussion

RBOH enzymes catalyze ROS production and play multiple critical roles in plant development, biotic and abiotic stress responses, signal transduction and hormone responses [25, 46–52]. Hence, *RBOH* genes have been widely identified in diverse plant species [39–44]. In this study, 44 putative *RBOH* genes were identified from six horticultural Rosaceae species; their numbers ranged from a minimum of 4 to a maximum of 10 in the Rosaceae genomes (Table 1). Among the *RBOH*s surveyed in a wider group of plants, the maximum number was found in the soybean genome (17), while the minimum number was found in sweet cherry (4). A comparison of *RBOH* numbers in 18 species (Fig 1A) indicated that the expansion of the *RBOH* gene family was not associated with an increase in the total number of predicted genes in a genome. We concluded that no direct relationship exists between genome size and the number of *RBOH* genes.

It has been reported that whole-genome duplication (WGD) may have had an important role in the expansion of the *RBOH* gene family in *Brassica rapa* because two gene pairs derived from a whole-genome triplication and three gene pairs derived from segmental duplications [39]. Whole-genome and segmental duplications occurred widely in the apple genome during the process of the apple domestication [53, 54]. As shown in Fig 2A, 8 of 10 *MdRBOH* genes were located on four homologous pair of chromosomes (chromosomes 6 and 14, 2 and 15, 3 and 11, 13 and 16) [54]. For example, chromosomes 2 and 15 are homologous pairs, and both contain an *MdRBOH* gene (named as *MdRBOH-E1* and *MdRBOH-E2*) (Fig 2A). Similar findings were also observed for chromosomes homologous pairs 13–16, 3–11, 6–14, and these genes were named as *MdRBOH-D1* and *MdRBOH-D2*, *MdRBOH-H* and *MdRBOH-J*, *MdRBOH-F1* and *MdRBOH-F2*. These findings clearly indicated that the duplication of these *MdRBOH* genes is related to apple whole-genome duplications. In addition, *MdRBOH-D1* and *MdRBOH-A* gene pairs was generated by segmental duplications. None of the *RBOHs* in the other five Rosaceae genomes appeared to have arisen from segmental duplication, probably because a WGD occurred during the process of apple domestication but did not occur in the other five species we investigated [53]. Notably, *RcRBOH-B1* and *RcRBOH-B2* appeared to be a tandem duplicated gene pair, based on a comprehensive analysis of their sequence properties and their genome locations.

Several studies have shown that ROS play a crucial role in the adventitious rooting process of herbaceous plants, such as cucumber, rice and *Chrysanthemum* [3, 18–20, 55]. However, the role of ROS in the adventitious rooting of woody plants has received less attention. In this study, exogenous H_2_O_2_ application significantly enhanced adventitious rooting of apple stem cuttings cultured in 1/2 MS medium supplemented with auxin (Fig 5). To our surprise, exogenous H_2_O_2_ treatment also rescued the adventitious rooting defect normally exhibited by apple stem cuttings grown on auxin-free 1/2 MS medium (Fig 5). It has been well established that auxin is necessary for adventitious root formation in apple [1, 6, 37]. To explain these observations, we speculate that auxin-induced adventitious root formation in apple is dependent upon on ROS production. Consistent with our inference, previous work has shown that exogenous NAA enhances ROS accumulation during adventitious rooting triggered by waterlogged conditions [3]. In addition, auxin-induced ROS are known to participate directly in root gravitropism, cell elongation, and quiescent center formation [56–58]. In our study, auxin-induced adventitious root formation was completely inhibited by preventing O_2_^−^ production using DPI (Fig 4). These results are supported by observations in cucumber seedling explants, in which ethylene-, ABA- or auxin-induced adventitious root formation was eliminated by DPI [3, 18, 59]. In addition, our findings that the amounts of O_2_^-^ accumulated mainly in the differentiating adventitious root primordia (Fig 6F), but almost undetected when adventitious root primordia were completely differentiated (Fig 6G) suggest that ROS are essential for adventitious root primordia induction. In agreement with our findings, O_2_^-^ production increased sharply after waterlogging inducing adventitious rooting in cucumber, and O_2_^-^ production also was elevated for only a short time [3]. Collectively, these results indicate that auxin-induced adventitious root formation is probably mediated by ROS. Hence, it will be interesting to learn the molecular basis of the crosstalk between auxin and ROS during the induction of adventitious root formation in woody plants.

Although ROS are clearly necessary for adventitious root formation, the functions of *RBOH* genes in adventitious rooting remain largely unexplored. It has been reported that the expression of *RBOH1* and *RBOH3* increased more than 2.5-fold during adventitious root formation triggered by waterlogging in wheat [4]. Similarly, the expression levels of *CsRBOHB* and *CsRBOHF3* were enhanced by ethylene and auxin, ultimately leading to adventitious root formation in cucumber [3]. In addition, *RBOHs* act downstream of auxin treatment in root hair elongation in rice and Arabidopsis [27, 28], and lateral root development in Arabidopsis [33]. In this study, the expression levels of *MdRBOH-A*, *MdRBOH-E1* and *MdRBOH-K* increased more than two-fold at day 9 after auxin treatment, coincident with the appearance of adventitious root primordia (Fig 7), indicating that these *RBOHs* may play a role in adventitious root formation. Based on the above results, we conclude that RBOHs act downstream of auxin to trigger ROS production in root hair, lateral root, and adventitious root development. However, the molecular link between auxin and *RBOHs* remain largely unknown. Until recently, the molecular link between auxin and ROS-mediated polar root hair growth has been established in Arabidopsis. They found that auxin-auxin response factors (ARF5) module to activation of the bHLH transcription factor RSL4, which directly regulates two *RBOHs* genes impacting on apoplastic ROS homeostasis, thereby stimulating root hair cell elongation [27]. In our study, *MdRBOH-E1* and *MdRBOH-K* were mainly expressed in the stem, whereas *MdRBOH-A* was highly expressed in both roots and stems (S3 Fig). Furthermore, analysis of *cis*-acting elements revealed that the *MdRBOH-E1* promoter contained an auxin-responsive element and the *MdRBOH-K* promoter contained a meristem expression element (S3 Table). Taken together, this evidence suggests that *MdRBOH-E1* and *MdRBOH-K* are candidates for the control of the adventitious rooting process. Further research will be needed to confirm their functions in adventitious root formation, and further experiments are required to identify the molecular link between auxin and *MdRBOH-E1* and *MdRBOH-K* genes expression in apple during adventitious rooting process.

## 5. Conclusions

The present study adds to our understanding of the evolutionary history of *RBOH*s by systematically analyzing their protein phylogeny, gene structures, conserved motifs, syntenic relationships, and expression patterns. We identified ten, four, seven, seven, seven and nine *RBOH* genes from apple, sweet cherry, almond, black raspberry and rose, respectively. These RBOHs were clustered into seven subfamilies that could be distinguished on the basis of their similar gene structures and conserved motifs. Interestingly, whole-genome duplications were found to be responsible for the expansion of the *RBOH* gene family in apple. The results of exogenous H_2_O_2_ and DPI treatment demonstrated that RBOH-derived ROS play an important role in adventitious root formation in apple. Expression analysis suggested potential functions of *MdRBOH-E1* and *MdRBOH-K* in adventitious rooting.

## Supporting information

S1 Fig Subcellular localization of MdRBOH-E1, MdRBOH-F1/F2, and MdRBOH-J. *GFP* was fused to the N terminus of *MdRBOHs* (MdRBOH-E1-GFP, MdRBOH-F1/F2-GFP and MdRBOH-J-GFP). The fluorescence signal of *N*.*benthamiana* leaves was detected by confocal microscope 72 hours after infiltration.
(TIF)

S2 Fig Protein alignment of all RBOH proteins. (TIF)

S3 Fig *MdRBOHs* expression in roots, leaves, stems, and shoot apex. Transcript levels were measured by qRT-PCR using apple *EF1α* as the reference gene. Data are means ± SD of three biological replicates. The statistical analysis was conducted using Duncan’s multiple range test (*P* < 0.05). The different lowercase letters indicate significant differences.
(TIF)

S1 Table Local blast results in order to identify the RBOHs. (XLSX)

S2 Table Conserved Domain analysis of candidate RBOHs. (XLSX)

S3 Table PCR primers of *MdRBOH* genes. (XLSX)

S4 Table Synteny analysis of *MdRBOH* and *AtRBOH* genes. (XLSX)

S5 Table Cis-elements in promoter region of *MdRBOHs*. (XLSX)
