# Supplementary figures and images for: Genome-wide identification and characterization of Respiratory Burst Oxidase Homolog genes in six Rosaceae species and an analysis of their effects on adventitious rooting in apple
Source: PLoS One. 2020 Sep 25;15(9):e0239705. doi: 10.1371/journal.pone.0239705 (PMC7518606; doi:10.1371/journal.pone.0239705)

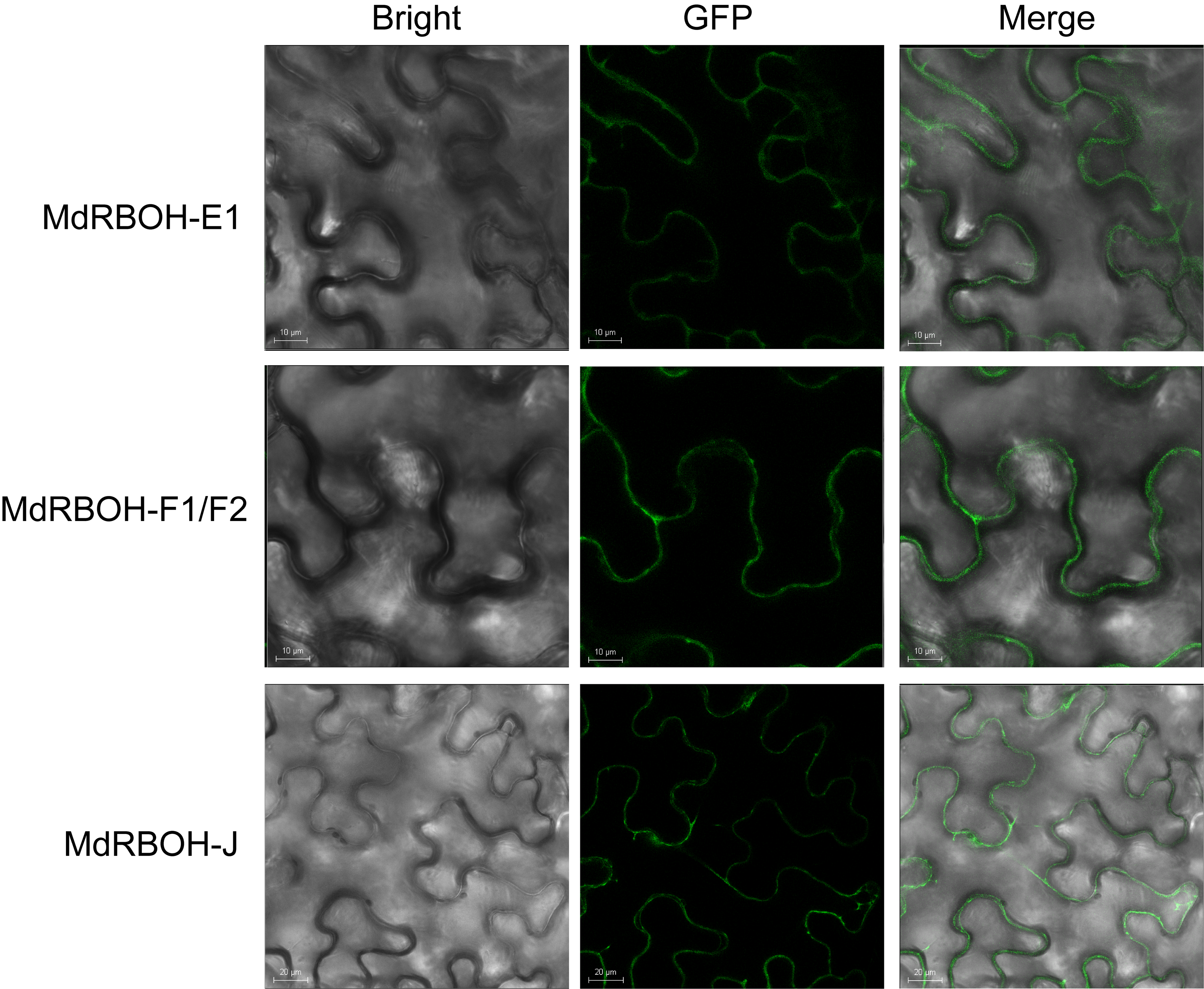

Supplement: S1 Fig — GFP was fused to the N terminus of MdRBOHs (MdRBOH-E1-GFP, MdRBOH-F1/F2-GFP and MdRBOH-J-GFP). The fluorescence signal of N.benthamiana leaves was detected by confocal microscope 72 hours after infiltration. (TIF) [file pone.0239705.s001.tif]

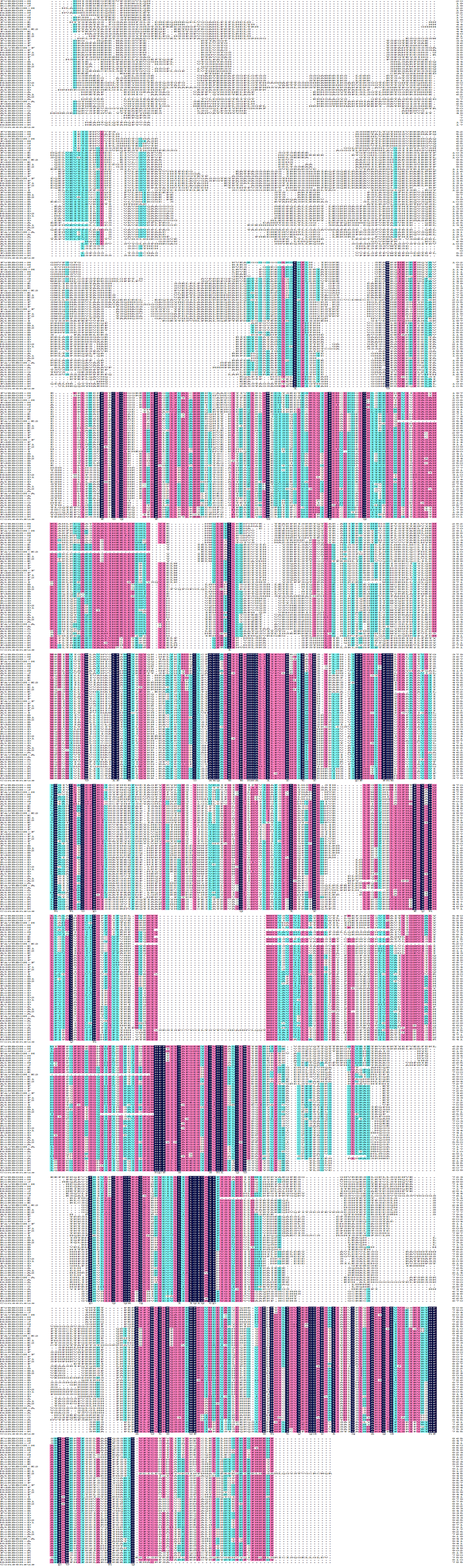

Supplement: S2 Fig — (TIF) [file pone.0239705.s002.tif]

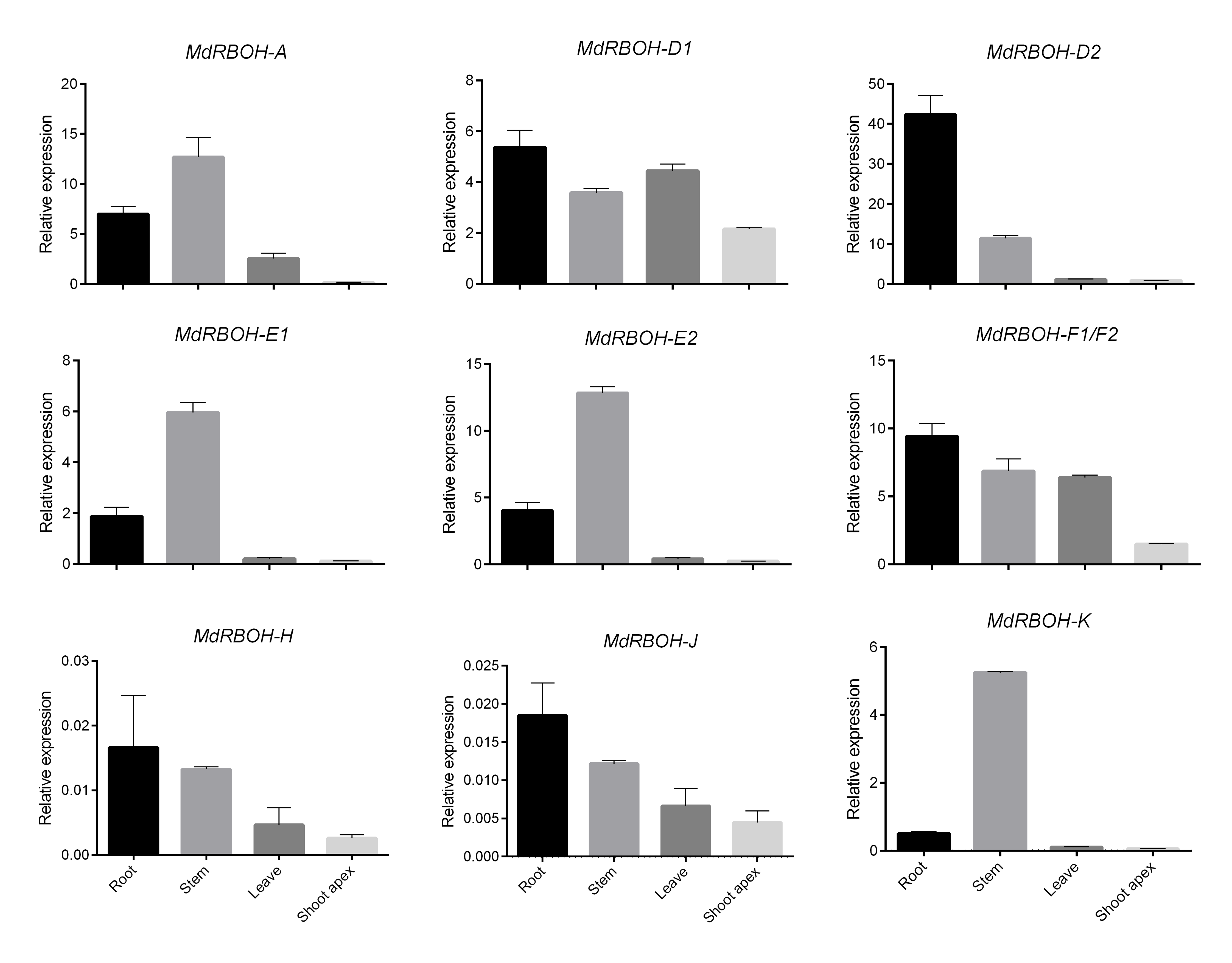

Supplement: S3 Fig — Transcript levels were measured by qRT-PCR using apple EF1α as the reference gene. Data are means ± SD of three biological replicates. The statistical analysis was conducted using Duncan’s multiple range test (P < 0.05). The different lowercase letters indicate significant differences. (TIF) [file pone.0239705.s003.tif]
